# Blood Cells as a Cellular Biomarker for Mitochondrial Function in a Experimental Model of Acute Carbon Monoxide Poisoning with Treatment

**DOI:** 10.1007/s13181-025-01077-6

**Published:** 2025-04-28

**Authors:** Devesh Bungatavula, John C. Greenwood, Frances S. Shofer, Guthrie Buehler, Shih-Han Kao, Matthew Kelly, Samuel S. Shin, Johannes K. Ehinger, Todd J. Kilbaugh, David H. Jang

**Affiliations:** 1 Drexel University, Philadelphia, PA USA; 2 Department of Emergency Medicine, Perelman School of Medicine, University of Pennsylvania, Philadelphia, PA USA; 3 Resuscitation Science Center (RSC), The Children’s Hospital of Philadelphia, Lab 814F 3615 Civic Center Blvd, Philadelphia, PA USA; 4 Department of Anesthesiology and Critical Care Medicine, Children’s Hospital of Philadelphia, Philadelphia, PA USA; 5 Department of Emergency Medicine, University of Alabama-Birmingham, Birmingham, USA; 6 Department of Neurology, Perelman School of Medicine, University of Pennsylvania, Philadelphia, PA USA; 7 Mitochondrial Medicine, Department of Clinical Sciences Lund, Lund University, Lund, Sweden

**Keywords:** Mitochondria, Blood Cells, Biomarker, Carbon Monoxide, Basic science

## Abstract

**Introduction:**

Carbon monoxide (CO) is a leading cause of environmental poisoning in the United States with substantial mortality and morbidity. The mechanism of CO poisoning is complex and includes hypoxia, inflammation, and mitochondrial dysfunction. Currently both biomarkers and therapies for CO poisoning are limited and require new approaches.

**Methods:**

Rats (~ 300 g) were divided into four groups of ten rodents per group (exposure): Control (room air), CO-400 (400 ppm), CO-1000 (1000 ppm) and CO-2000 (2000 ppm). Rodents received the assigned exposure through a secured tracheotomy tube over 120 min followed by 30 min of re-oxygenation at room air for a total of 150 min. Five additional rodents in each group were administered a succinate prodrug (NV354) at the start of exposure for the duration of the experiment until the reoxygenation period as separate experiments. Cortical brain tissue and whole blood were obtained for mitochondrial respiration. Stored plasma and snap frozen tissue stored at -80^o^C were used to obtain protein quantification with Western Blotting.

**Results:**

All animals in the Sham, CO-400, and CO-1000 groups survived until the end of the exposure period; no animals in the CO-2000 groups survived the exposure and were counted as attrition. We observed a dose-dependent decrease in key respiratory states in both isolated brain mitochondria and peripheral blood mononuclear cells (PBMCs), and, PBMCs respiration more positively correlated with isolated brain mitochondria when compared to carboxyhemoglobin (COHb). There was no significant difference in mitochondrial respiratory states in animals treated with NV354 compared to the untreated group.

**Conclusions:**

The primary findings from this study include: (1) A dose-dependent decrease with key respiration states with higher concentrations of CO; (2) PBMCs had a higher correlation to isolated brain mitochondria respiration when compared to COHb; and (3) there was no treatment effect with the use of NV354.

**Supplementary Information:**

The online version contains supplementary material available at 10.1007/s13181-025-01077-6.

## Introduction

Carbon monoxide (CO) is a colorless and odorless gas that is an important cause of poisoning mortality and morbidity in the United States with approximately 15,000 intentional cases annually, accounting for 400–500 reported deaths each year. Specifically, there are over 50,000 CO cases seen in emergency departments in the US annually, with over half requiring hospitalization [1]. It is estimated that CO poisoning results in over $1 billion annually in hospital costs and lost earnings [2]. The most serious complications for survivors of consequential CO exposure are neurologic failure with delayed neurological sequelae (DNS) occurring in up to 50% of patients [3, 4]. While hypoxia, inflammation and lipid peroxidation are known to play a part in the complex mechanism of CO poisoning, another important mechanism of injury is mitochondrial dysfunction [5–7]. CO targets cytochrome *c* oxidase, also known as Complex IV (CIV), resulting in bioenergetic failure and propagation of reactive oxygen species (ROS) leading to organ dysfunction.

The mitochondria are involved in essential functions beyond energy production and may be the final regulator determining cellular fate following injury and poisoning [8, 9]. Mitochondria play a significant role in cellular metabolism where oxygen (O_2_) consumption through the mitochondrial electron transport system (ETS) is tightly coupled to ATP production and is regulated by metabolic demand. Failure of the mitochondria to use O_2_ to sustain ATP production results in an energy deficit that can impair cell function [10, 11]. Decreased ETS activity and low ATP levels in muscle biopsies have been linked to death in other forms of shock such as sepsis and traumatic injuries [12–14]. Our own work has indicated the vital role that the mitochondria have in the pathophysiological process after acute CO poisoning, using our unique swine platform to obtain advanced physiological and biomolecular outcomes in this study [15–17].

At this time there is a lack of adequate biomarkers to gauge the severity of CO poisoning as well as inability to single out those patients who may develop serious neurologic morbidity and to monitor response to treatment. Current diagnosis of CO poisoning relies on history and a carboxyhemoglobin (COHb) level, however, COHb levels have poor correlation with severity and do not reflect the degree of organ dysfunction often delaying diagnosis and treatment. Thus, there is an urgent need for reliable, informative markers of early mitochondrial dysfunction associated with CO poisoning as these could enable early intervention in the subclinical stages of disease and direct therapy [18]. Blood cells has been used as biomarkers of mitochondrial function in a wide range of clinical disease ranging from cardiac arrest, sepsis, traumatic hemorrhage, and poisoning. Ex vivo measurement of mitochondrial function in blood cells obtained from patients with confirmed CO poisoning show promise to predict severity of cellular injury from CO [19–23].

Another objective for this study was to investigate a novel succinate prodrug (NV354) in our rodent model of acute CO poisoning. The current standard of care for CO poisoning consists of supportive care and use of hyperbaric oxygen (HBO). While these current treatments may mitigate symptoms and reduce immediate mortality, survivors are still at risk for developing cognitive and motor function deficits well after the acute CO symptoms have subsided. To address this critical gap in therapy, our team has developed a library of novel, plasma stable succinate prodrugs, whose intracellular cleavage yields bioavailable succinate, which in turn increases ATP-linked respiration at Complex II (CII) [24–27]. The drug does not act on CO itself but may compensates for cellular injury to prevent organ failure.

The objectives of this study were to: (1) investigate the dose-dependent effect of CO exposure on mitochondrial function in the brain using a rodent model; (2) to evaluate the ability of blood cell respirometry to act as a biomarker for CO severity; and (3) to evaluate the effect of a mitochondrially targeted therapeutic.

## Materials and methods

### Animals and Overall Study Design

This was a non-survivor rodent model of acute CO poisoning to investigate cerebral mitochondrial dysfunction and use of blood cells as a biomarker. An equal number of female and male Sprague-Dawley (Charles Rivers Laboratories, Wilmington, MA, USA) rats weighing ~ 300 g were used for this study. All rodents were placed in an induction chamber with 5% isoflurane exposure for 1–2 min and upon induction placed on a SurgiSuite Multi-Functional Surgical Platform (Kent Scientific, Torrington, CT, USA) with warming capacity and placement of a rectal temperature sensing probe to maintain a core temperature of 37–38^o^C. The animals were randomized into four different groups: Sham (*n* = 10), CO-400 (*n* = 10), CO-1000 (*n* = 10), and CO-2000 (*n* = 10) with the last three groups referring to the severity of the acute CO exposure. An additional 5 subject animals for each group underwent their assigned exposure but were treated with our succinate prodrug. All procedures were approved by the Institutional Animal Care and Use Committee at the Children’s Hospital of Philadelphia and performed in accordance with the National Institutes of Health Guide for the Care and Use of Laboratory Animals.

### Perioperative Procedures and Monitoring

After induction, all subjects were placed on the surgical platform and a rodent cone was used (2–3% isoflurane) to maintain general anesthesia (tail and toe pinch used to assess depth of anesthesia) for the described procedures. Immediately upon verification of anesthesia, all subject animals underwent immediate tracheostomy and were placed on a small animal ventilator (VentElite Small Animal Ventilator from Harvard Apparatus, Holliston, MA, USA). Ventilator settings were as follows on pressure support: respiratory rate of 50–55 breathes/min and a pressure of 8–10 to maintain an end-tidal carbon dioxide of 35–45 mmHg. Isoflurane was weaned to approximately 1–1.5% to simulate human anesthetic protocols and minimize confounding toxicity associated with higher doses of isoflurane. The right carotid was dissected out with isolation of the carotid from the internal jugular vein and vagal nerve under 10x magnification. Upon isolation of the carotid artery, a Millar SPR-869 Rat PV Catheter (2 F, 4E, 6 mm, 15 cm, PI) was advanced into the left ventricle of the heart and pressure-loop analysis was obtained. All data were recorded with PowerLab 16/35 LabChart 8 Pro software from ADInstruments (Sydney, Australia) [24, 28, 29].

### Carbon Monoxide Experimental Protocol

For this study we utilized three different CO concentrations to investigate dose dependent effects on our proposed biomolecular measures. We specifically used a dose of 400 ppm as mild, 1000 ppm as moderate and 2000 ppm as severe exposure. CO was administered from a CO tank 244 cf. using a Regulator 0–10 L/min with flow meter from Airgas (Radnor Township, PA, USA). The CO concentration was monitored using an Inspector Carbon Monoxide Detector 0–2000 ppm range (Sensorcon, New York, USA) integrated into the tubing to directly monitor the CO tank output. Animals received their assigned CO exposure for 120 min followed by 30 min of room air to mimic removal of subjects from a CO exposure. Animals in the Control group received 150 min of room air. All animals were maintained on inhaled isoflurane (~ 1%) for the duration of the procedure. COHb was measured with a ABL90 FLEX PLUS blood gas analyzer (Radiometer America, California, USA).

### NV354 Protocol (succinate prodrug)

NVP354 is a succinate prodrug (Abliva AB, Stockholm, Sweden) developed for the treatment of rare and severe primary mitochondrial disease. We administered NV354 as a single, IV bolus dose at 17 mg/kg followed by continuous venous infusion at 25 mg/kg/h for 180 min. The dose selection was based on previous data, indicating lack of side-effects of the given dose in several different species, and efficacy when dosed orally in a rat rotenone disease model [24, 30].

### Tissue and Whole Blood Extraction and Preparation

Upon completion of the protocol described above, all subject animals were euthanized after which the brain and whole blood were immediately obtained. 4 mL of whole blood was obtained through cardiac puncture for both plasma and blood cells for mitochondrial respirometry. Using a combination of 15 mL of Ficoll-Paque^™^ PLUS, a 50 mL Leucosep tube (Greiner Bio-one) and centrifugation, a population of peripheral blood mononuclear cells (PBMCs) from a buffy coat. Brain tissue immediately underwent rapid but gentle dissection and were then immediately transferred into ice-cold buffer solution (320 mM sucrose, 2 mM EGTA, 10 mM Trizma base, pH 7.4). Next, brain tissue was minced, manually homogenized in ice-cold brain buffer (225mM D-Mannitol, 75mM Sucrose, 5mM HEPES, 1mM EGTA and 0.5 L of double deionized water, pH 7.4) containing 0.2% BSA buffer (catalog A6003) and centrifuged at 1300 g and 4 °C to discard cell debris. Subsequently, the supernatant was centrifuged for 10 min at 21,000 g to extract the mitochondrial pellet. Brain mitochondria were further isolated from the derived pellet by differential centrifugation and application of density gradients using 15%, 23% and 40% Percoll (GE Healthcare cat. no. 17089101), washed with brain buffer, and centrifuged to collect the isolated mitochondrial pellet. Protein count for isolated mitochondria was obtained with a Pierce BCA Protein Assay kit (catalog 23227) from Thermo Fisher Scientific (Waltham, MA, USA). In general, the time from tissue acquisition to completion of protein quantification for mitochondrial respiration was approximately 2.5 h which is well within mitochondrial viability with this established protocol [15, 19, 24, 28, 30].

### Measurement of Mitochondrial Respiration

Mitochondrial respiratory function was analyzed using multiple Oroboros O2k-FluoRespirometers (Oroboros Instruments, Innsbruck, Austria) with a substrate–uncoupler–inhibitor titration (SUIT) protocol as previously described in our work [31–33]. The SUIT protocol measures oxidative phosphorylation capacity with electron flow through Complex I (CI) and Complex II (CII) respectively, as well as the convergent electron input (CI + CII) using the nicotinamide adenine dinucleotide-linked substrates, malate (5 mM) and pyruvate (5 mM) and glutamate (5 mM), as well as the flavin adenine dinucletodide-linked substrate succinate (10 mM), both in the presence of adenosine diphosphate (1 mM). Oxidative phosphorylation produces adenosine triphosphate (ATP), which is the primary fuel for performing basic function. During times of stress and injury, increased maximal oxidative phosphorylation is necessary for neuronal salvage and repair because of high energy requirements. Oligomycin, an inhibitor of the ATP-synthase, uncouples respiration from ATP-synthase activity to measure respiration where the O_2_- consumption is dependent on the leakiness the mitochondrial membrane and back-flux of protons into the mitochondrial matrix that is independent of the ATP synthase (LEAK_CI+CII_). If LEAK respiration is increased, the electrochemical gradient across the mitochondrial membrane is uncoupled, resulting in inadequate ATP production. Maximal convergent non-phosphorylating respiration of ETS_CI+CII_ is evaluated by titrating the protonophore, carbonyl cyanide p-(trifluoromethoxy) phenylhydrazone. ETS_CI+CII_ can be considered a stress test for mitochondria, as a marker of mitochondrial respiratory reserve. Non-phosphorylating respiration through CII (ETS_CII_) is achieved through the addition of rotenone (2 mM). The Complex III (CIII) inhibitor antimycin-A (5 mM) is added to measure the residual non-mitochondrial oxygen consumption, and this value was subtracted from each of the measured respiratory states to provide only mitochondrial respiration. Complex IV (CIV)-linked respiration was measured by the addition of N,N,N´,N´-tetramethyl-phenylenediamine (0.5 mM) together with ascorbate (0.8 mM). The CIV inhibitor sodium azide (10 mM) was added to reveal the chemical background that is subtracted from the N,N,N’,N’-tetramethyl-phenylenediamine-induced oxygen consumption rate. When measuring respiration in PBMCs, we used a controlled permeabilization with digitonin (1 µL/1 × 10^6^ cells) to allow passage of substrates to the mitochondria. Isolated mitochondria from the brain cortex were normalized to protein count and PBMCs were normalized to cell count (per 10^6^ cells) obtained and protein content (as described above). All respirometry data were acquired using DatLab 7 (Oroboros Instruments, Innsbruck, Austria).

### Western Blotting

Western blot was performed on tissue with all reagents and antibodies purchased from Invitrogen (Carlsbad, CA, USA) unless otherwise noted. Cortical brain tissue was homogenized based on our previous methods for Western blotting [15, 28, 34]. Equal protein content was loaded into each well of a 12% Bis-Tris gel and separated by sulfate-polyacrylamide gel electrophoresis (SDS-PAGE). Gel proteins were transferred onto a nitrocellulose membrane (Catalog IB23001) and then incubated with a citrate synthase (CS) recombinant rabbit monoclonal antibody (Catalog 3H8L26) with dilution factor of 1:2000 in the iBind solution (Catalog SLF1020). Primary rabbit monoclonal anti-glyceraldehyde 3-phosphate dehydrogenase (GAPDH) antibody conjugated to horseradish peroxidase (HRP) (#3683 Cell Signaling Technology; 1:2000) was used as an internal control. CS levels were detected using a goat anti-rabbit IgG secondary antibody conjugated to HRP (catalog A16096; 1:400) and a chemiluminescent substrate reagent kit. Upon completion of the detection step using a chemiluminescent substrate reagent kit (Invitrogen Catalog 34579), imaging was performed with an iBright Analysis Software (Thermo Scientific) to obtain the quantification and densitometric analysis of the blots. All experiments were performed in duplicates (each subject was done in duplicate across two different gels) and the local background corrected density values was normalized against their respective housekeeping proteins described above.

### Statistics and Data Analysis

To determine differences in rodent characteristics and venous blood gases at 150 min, analysis of variance (ANOVA) was used. Results for these characteristics are presented as mean ± standard deviation. To determine differences in respiration between CO groups for isolated brain mitochondria, PBMCs, and citrate synthetase, Kruskal-Wallis tests were performed. To adjust for multiple comparisons after a significant test, post-hoc pairwise comparisons using Dunn’s test were performed. Results are presented as median and interquartile range (IQR). To examine whether PBMCs respiration or COHb could predict isolated brain mitochondrial respiration, a linear regression was used. All analyses were performed using SAS statistical software (version 9.4, SAS Institute, Cary NC). Figures were created using GraphPad Prism (version 10.4.1, GraphPad Software, San Diego, CA, USA).

## Result

### Baseline Characteristics, Blood Gas Chemistry, Hemodynamic Variables

The baseline weight, blood gases, and COHb obtained at the start of the experiment were similar between animals (data not shown). All animals in the Sham, CO-400, and CO-1000 group survived for the duration of the exposure. There were no animals that survived until the end of the exposure in the CO-2000 group and hence this group was not included in the final analysis. Serial venous blood gases were obtained every 30 min along with continuous vital sign monitoring throughout the experiment [Table 1].

**Table 1:** The variables are at the 150 min time point that includes 120 min of exposure and 30 min of re-oxygenation. There were no survivors in the CO-2000 group. These were counted as attrition and not included in the analysis. Values presented as mean ± standard deviation

	Sham	CO-400	CO-1000	Sham ― CO-400		Sham ― CO-1000		CO-400 ― CO-1000	
	(*n* = 10)	(*n* = 10)	(*n* = 10)	D	p-value	D	p-value	D	p-value
Heart rate (BPM)	305.2 ± 4.8	315.6 ± 6.1	325.4 ± 5.4	-10.4	***0.0006***	-20.2	***< 0.0001***	-9.8	**0.0012**
Systolic (mmHg)	100.2 ± 6.9	99.3 ± 5.7	86.6 ± 7.6	0.9	0.95	13.6	***0.0003***	12.7	**0.0007**
Diastolic (mmHg)	55.3 ± 4.6	55.4 ± 5.1	44.6 ± 4.6	-0.1	> 0.99	10.7	***< 0.0001***	10.8	**< 0.0001**
MAP (mmHg)	70.3 ± 4.7	70.2 ± 4.6	58.4 ± 5.3	0.1	> 0.99	11.9	***< 0.0001***	11.7	**< 0.0001**
*Blood gases (venous)*									
pH	7.4 ± 0.1	7.4 ± 0	7.3 ± 0	0.006	0.96	0.084	***0.0012***	0.078	**0.0025**
PCO_2_ (mmHg)	41 ± 2.6	39.9 ± 3.1	33.7 ± 3.1	1.1	0.68	7.3	***< 0.0001***	6.2	**0.0002**
HCO_3_ (mmol/L)	24.1 ± 1.5	23.2 ± 1.2	22.3 ± 1.1	0.9	0.277	1.8	***0.0112***	0.9	0.277
Lactate (mmol/L)	1.2 ± 0.2	1.2 ± 0.1	1.6 ± 0.3	-0.02	0.98	-0.41	***0.001***	-0.39	**0.0016**
COHb	0.6 ± 0.5	9.7 ± 2.4	29.9 ± 7.9	9.1	***0.0006***	-29.3	***< 0.0001***	-20.2	**< 0.0001**

### Mitochondrial Respiration

**Fig. 1:**
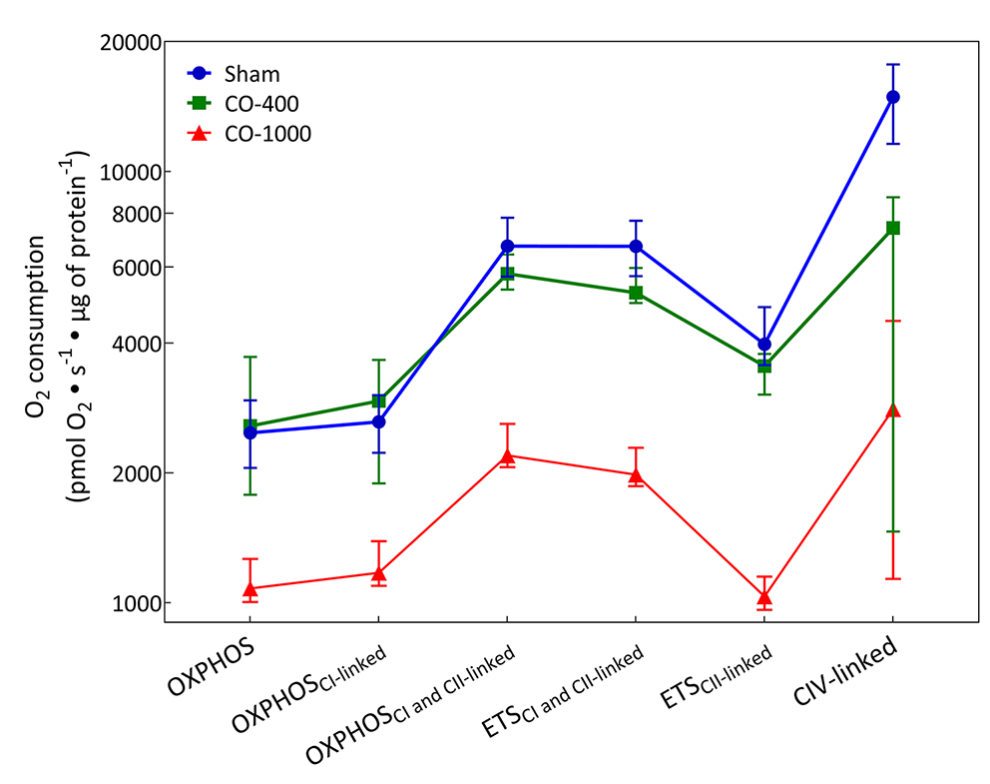
Respiration in Isolated Brain Mitochondria. The oxygen consumption of isolated mitochondria from cortical brain tissue was measured ex vivo at the conclusion of the animal experiment. All values are represented as median (IQR). OXPHOS: oxidative phosphorylation; ETS electron transport system; C: Complex

**Fig. 2:**
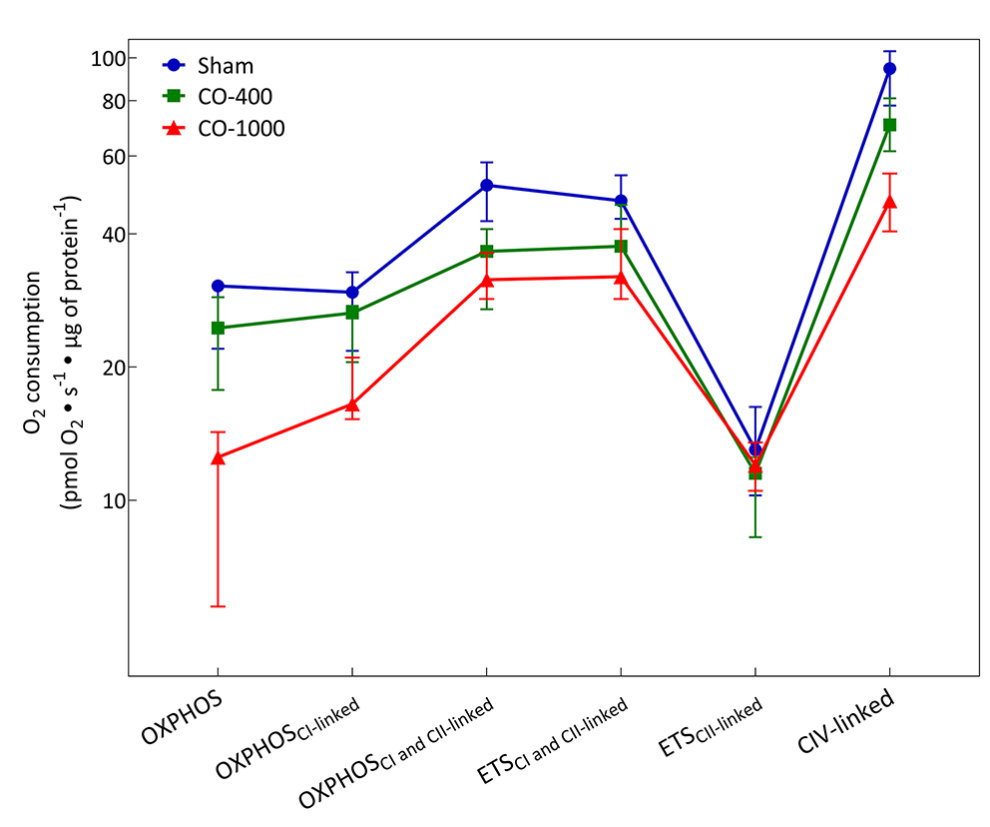
Respiration in PBMCs. The oxygen consumption of PBMCs was measured ex vivo at the conclusion of the animal experiment. All values are represented as median (IQR). OXPHOS: oxidative phosphorylation; ETS: electron transport system; C: Complex; PBMC: Peripheral blood mononuclear cell

The oxygen consumption of isolated mitochondria from brain tissue [Fig. 1] and PBMCs [Fig. 2] were measured ex vivo at the conclusion of the experiment. For the isolated brain mitochondria, the results were as follows for the respiratory states: (1) Sham vs. CO-400: There was no significant difference between groups except at CIV-linked respiration where Sham was higher; (2) Sham vs. CO-1000: The Sham group had significantly higher values for all respiratory states; (3) CO-400 vs. CO-1000: The CO-400 group had significantly higher values for all respiratory states except CIV-linked. For PBMCs, there was no difference between groups for the respiratory state with ETS_CII_ and all groups differed from each other for CIV-linked. Additionally, for (1) Sham vs. CO-400, the Sham group had significantly higher values at OXPHOS_CI+CII-linked_ and ETS_CI+CII_; (2) Sham vs. CO-1000: The Sham group had significantly higher values for OXPHOS, OXPHOS_CI-linked_, OXPHOS_CI+CII-linked_ and ETS_CI+CII_ respiratory states;; (3) CO-400 vs. CO-1000: The CO-400 group had significantly higher values for OXPHOS and OXPHOS_CI-linked_. [Supplementary Table 1].

### Blood Cells as a Biomarker

**Fig. 3:**
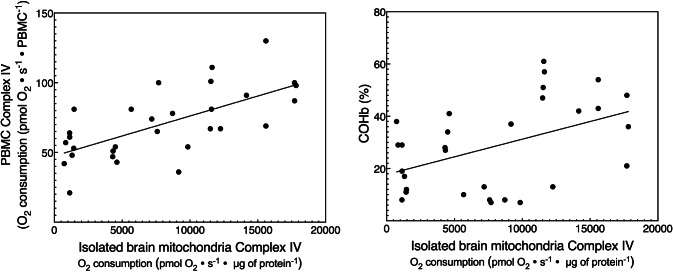
PBMC (CIV respiration) versus COHb as a biomarker for brain mitochondrial dysfunction. The correlation coefficient for PBMC CIV respiration against isolated brain mitochondrial CIV respiration was *r* = 0.66 which was higher than the use of COHb which had a correlation coefficient of *r* = 0.45

PBMC CIV respiration was more highly correlated (*r =* 0.66) with isolated brain mitochondrial CIV respiration compared to COHb (*r* 0.45, Fig. 3).

### Succinate Prodrug

**Fig. 4:**
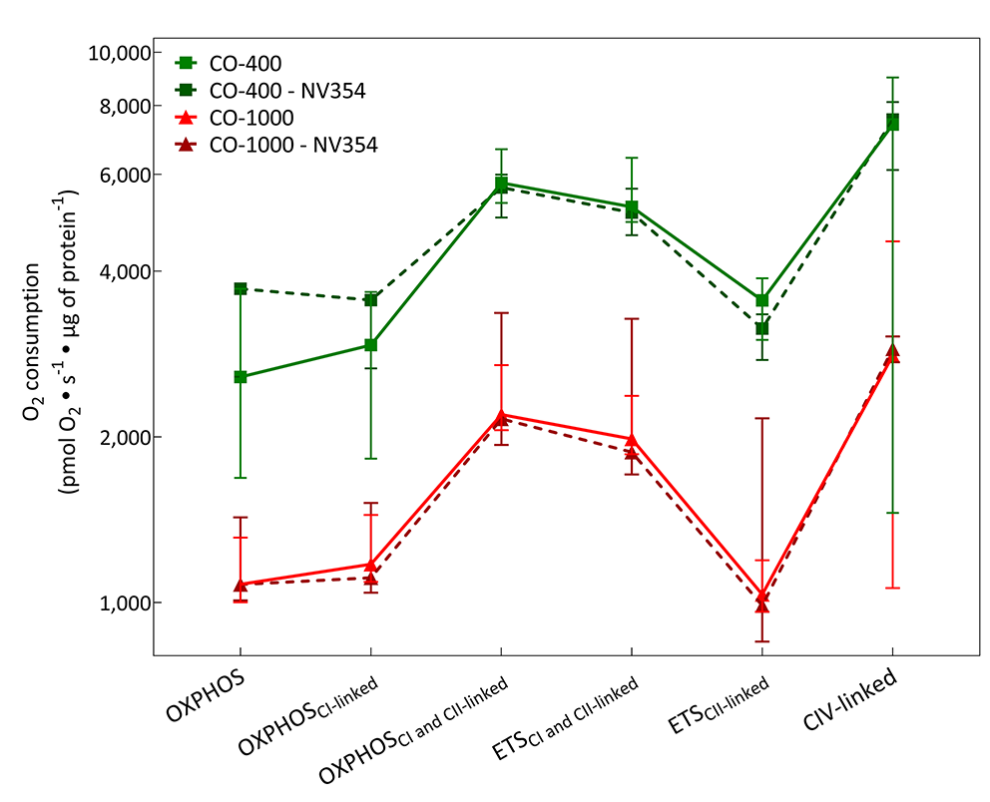
Isolated Brain Mitochondrial Respiration with NV354. The oxygen consumption of isolated brain mitochondria from brain tissue was measured at the conclusion of the experiment in animals treated with NV354 during their assigned CO exposure compared against their respective untreated CO exposure. There was no significant difference between the treated and untreated animals across all key respiratory states. All values are represented as median (IQR)

For each group an additional five animals received NV354 as a therapy for acute CO poisoning. All animals that received NV354 survived until the end of exposure for tissue collection in the Sham, CO-400, and CO-1000 group. There was no significant difference in respiration of isolated brain mitochondrial of the animals that were treated with NV354 when compared to their respective exposure group [Fig. 4].

### Western Blot

**Fig. 5:**
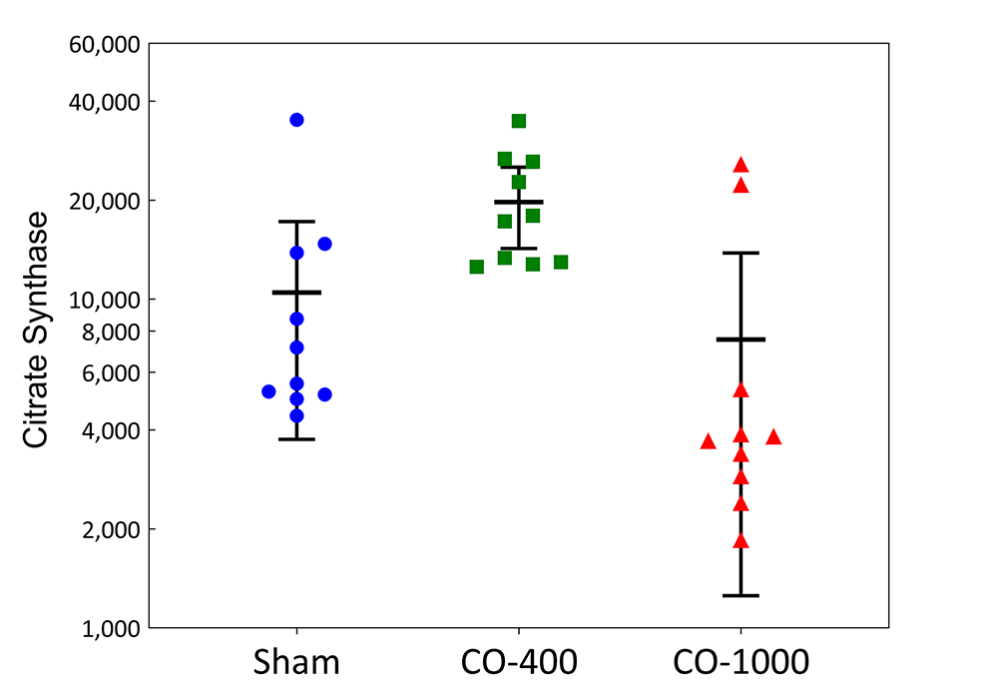
Western blot for citrate synthase. The protein expression of citrate synthase (a marker of mitochondrial content) of the CO-1000 group was significantly lower than the CO-400 group. All values are represented as median (IQR)

The relative density of citrate synthase (CS) in snap-frozen cortical brain tissue were compared between the three groups. The CS relative density was 6332 (5094–14081), 17,569 (12918–26338), and 3773 (2775–9583) for the Sham, CO-400, and CO-1000 groups, respectively. The only significant difference was between the CO-400 and the CO-1000 group, *p* < 0.01 [Fig. 5].

## Discussion

The objective of this study was to use our advanced rodent model of CO poisoning to evaluate the use of PBMCs as a potential biomarker of disease severity and cerebral mitochondrial function following exposure to different doses of CO that mimics clinical reality. We also tested our succinate prodrug (NV354) as a possible therapeutic for acute CO poisoning to potentially attenuate mitochondrial dysfunction. An overall key finding was the demonstration of cerebral mitochondrial dysfunction in a dose-dependent fashion as well as the potential of blood cells as a biomarker.

One of the strengths of this study was the ability to study clinically relevant and discrete doses of CO. Using our advanced rodent platform, we were also able to obtain real-time physiological metrics used in clinical medicine such as heart rate and blood pressure along with serial blood gases to assess acid-base disturbances that helps to inform the interaction of the physiological changes to the biomolecular measures described in this study. With increasing doses of CO from room air to 1000 ppm there were significant increase in heart rate and changes in hemodynamics with a corresponding increase in the COHb concentration with the higher CO doses. It is interesting to note that there were no survivors in the CO-2000 group. This is not surprising given that an acute exposure of 2000 ppm is severe and often fatal with prolonged exposure. It is also important to note that there are clear species difference in the response to acute CO exposure as our experience in swine models show that many of our swine can tolerate over 3 h of CO at 2000 ppm. This difference may be related to the body surface area and higher metabolic rate as smaller animals such as rodents or birds may be more sensitive to the adverse effects of CO [15–17, 35].

An important finding from this study is the effect of CO on the mitochondria. Our previous studies have strongly implicated the critical role the mitochondria have in CO poisoning using both clinical samples and experimental models [22, 33, 34]. The measurement of respiration has been considered a gold standard in studying mitochondrial physiology. Using three clinically relevant doses, we further investigated the vital role of the mitochondria using a dose-response. In this study we found that with increasing doses of CO there was a corresponding decrease in overall mitochondrial function, at CIV which is a major site of action of CO. This relationship was also observed in the PBMCs obtained in the same group of animals, which also demonstrated a dose dependent effect on mitochondrial function, also at CIV [19, 36, 37].

One of the primary objectives of this study was to evaluate the application of blood cells using PBMCs as a biomarker of acute CO poisoning using a non-survivor rodent model. In this study we found that when PBMCs were used as a surrogate for brain mitochondrial function, it had a higher correlation when compared to the use of COHb. Currently COHb serves as a clinical marker of exposure. Several studies have shown COHb to be a poor marker of disease severity and thus there is a need for more sensitive biomarkers. One easily accessible tissue that has been investigated in various disease states are blood cells that may act as a “window” to various target organs as they are non-adherent cells that circulate throughout the body. The application of blood cells as biomarkers in acute poisoning is currently limited [19, 36, 37]. Our group has shown that ex vivo blood cell respirometry appears to be a more sensitive biomarker of CO poisoning in clinical samples when compared to a COHb.

This preclinical study suggests the potential application of blood cells as a biomarker for CO poisoning, but there are some important considerations for possible clinical application. One of the potential barriers is the amount of time to perform the experiment to obtain mitochondrial respiration in blood cells. The time from whole blood draw to PBMC isolation and eventual respiration data was about 2.5 h. Our earlier works in this area focused on blood cells from patients with confirmed CO poisoning to measure PBMC respiration for use as a possible biomarker. While this does require specialized equipment and skillset to perform, continued work in this area and technology development for a point-of-care device would make clinical application more feasible in the future.

Another important objective of this study was also to test our succinate prodrug (NV354) to assess any reversal of mitochondrial dysfunction imposed by CO. Our compound is a succinate prodrugs that permeate the cell membrane to deliver succinate to the mitochondria, thus increasing respiration through the CII pathway. While primarily designed to bypass CI dysfunction, our prior in vitro work has shown improved mitochondrial function in a wide range of CIV poisons such as cyanide and CO. While CIV inhibition may theoretically result in downstream blockade to the increased CII respiration by the succinate prodrug, our prior results for this application is likely due to incomplete CIV inhibition as total CIV inhibition is incompatible with eucaryotic life. In this study we used both the dose and duration of NV354 based on our prior publications in this area mostly related to application with organophosphate (CI inhibitor) and the lack of rescue effect may be related to both dose and duration that warrant further work in this area [24, 26, 27, 30]. The plasma half-life of NV354 is also markedly lower in rodents compared to humans and swine, and this approach needs to be tested in another model system for any final conclusions about its applicability to be drawn.

We also measured CS protein density across the three groups using western blotting. CS is a pace-maker enzyme in the Krebs cycle localized in the mitochondrial matrix, with a molecular weight of 85 kDa. It is often used as a measure of mitochondrial content in tissues and cellular samples. In this study there was a significant decrease in CS expression in the CO-1000 group when compared to the CO-400 group, a likely contributor to the decreased respiration. However, as all measures of mitochondrial respiration weren’t equally affected, there is a qualitative reduction in CIV specifically, and not just a quantitative reduction in mitochondria, as that would be equally reflected along more respiratory states.

There are limitations to consider in this study. While these results are encouraging for the clinical application of blood cell respirometry as a disease severity biomarker in patients with CO poisoning, some known limitations include the heterogenous patient population that could make extrapolations to other populations more difficult. While these studies attempted to control for various factors such as medical history, it is known underlying medical conditions such as hypertension and diabetes can result in altered mitochondrial function in blood cells. An important limitation in clinical studies is the varied CO exposure. One of the strengths of this current study was controlling for both the dose and duration of CO exposure using homogenous subjects to better control for variability using blood cell respirometry as a biomarker. Another important consideration is that this was a non-survivor study using rodents. We have also performed CO experiments using swine that better approximate human physiology and survivor studies that will allow future studies in this area.

## Conclusions

There was a dose-dependent decrease in key respiration states with higher concentrations of CO, and PBMC respiration had a higher correlation to isolated brain mitochondria respiration when compared to COHb. Finally, there was no treatment effect on mitochondrial respiration with the use of NV354.

## Electronic Supplementary Material

Below is the link to the electronic supplementary material.

Supplementary Material 1

## Data Availability

All original data and materials are kept in a locally managed OneDrive secured database at the Children’s Hospital of Philadelphia (CHOP).
