# Supplementary material for: Blood Cells as a Cellular Biomarker for Mitochondrial Function in a Experimental Model of Acute Carbon Monoxide Poisoning with Treatment
Source: J Med Toxicol. 2025 Apr 28;21(3):327–35. doi: 10.1007/s13181-025-01077-6 (PMC12204972; doi:10.1007/s13181-025-01077-6)
Supplement: Supplementary file 1 — Supplementary Material 1 [file 13181_2025_1077_MOESM1_ESM.docx]

**Supplementary Table 1 – Respiration Values**

| Mitochondrial respiration (pmol O_2_ • s^-1^ • µg of protein^-1^) | | | | | | |  | |  | |  | | |  | | | |  | | | |  | | | |  | | |  |
| --- | --- | --- | --- | --- | --- | --- | --- | --- | --- | --- | --- | --- | --- | --- | --- | --- | --- | --- | --- | --- | --- | --- | --- | --- | --- | --- | --- | --- | --- |
| **Isolated Brain Mitochondria** | **Sham** | | | | **CO-400** | | | **CO-1000** | | | | **p-value** | | | | **Sham vs CO-400** | | | | **Sham vs CO-1000** | | | | **CO-400 vs CO-1000** | | |  |  |  |
| OXPHOS | 2477 | (2054-2946) | | | 2569.5 | (1779-3715) | | 1079 | | (1004-1263) | | | 0.0016 | | | | 0.4899 | | | | ***0.0001*** | | | | ***0.0002*** | |  |  |  |
| OXPHOS_CI-linked_ | 2626 | (2226-3030) | | | 2936 | (1890-3656) | | 1173.5 | | (1095-1389) | | | 0.0001 | | | | 0.4394 | | | | ***0.0015*** | | | | ***0.0008*** | |  |  |  |
| OXPHOS_CI+CII-linked_ | 6708 | (5706-7810) | | | 5789.5 | (5322-6416) | | 2195 | | (2062-2599) | | | 0.0001 | | | | 0.1064 | | | | ***<.0001*** | | | | ***0.0008*** | |  |  |  |
| ETS_CI+CII_ | 6704 | (5719-7687) | | | 5233.5 | (4955-5972) | | 1982 | | (1862-2286) | | | 0.0001 | | | | 0.1066 | | | | ***<.0001*** | | | | ***0.0011*** | |  |  |  |
| ETS_CII_ | 3973.5 | (3556-4848) | | | 3539 | (3039-3774) | | 1034.5 | | (963-1149) | | | 0.0001 | | | | 0.0891 | | | | ***<.0001*** | | | | ***0.0013*** | |  |  |  |
| CIV-linked | 14881.5 | (11583-17709) | | | 7389.5 | (1462-8711) | | 2809.5 | | (1136-4502) | | | 0.0083 | | | | ***0.0017*** | | | | ***<.0001*** | | | | 0.0703 | |  |  |  |
|  | | |  |  | | |  | |  | |  | | |  | | | |  | | | |  | | | |  | | |  |
| Mitochondrial respiration (pmol O_2_ • s^-1^ • µg of protein^-1^) | | | | | | |  | |  | |  | | |  | | | |  | | | |  | | | |  | | |  |
| **PBMCs** | **Sham** | | | | **CO-400** | | | **CO-1000** | | | | | **p-value** | | **Sham vs CO-400** | | | | **Sham vs CO-1000** | | | | **CO-400 vs CO-1000** | | | | |  |  |
| OXPHOS | 30.5 | (24-31) | | | 24.5 | (18-28) | | 12.5 | | (6-14) | | | 0.0003 | | 0.2307 | | | | ***<.0001*** | | | | ***0.0011*** | | | | |  |  |
| OXPHOS_CI-linked_ | 29.5 | (22-32) | | | 26.5 | (21-27) | | 16.5 | | (16-21) | | | 0.0001 | | 0.2385 | | | | ***0.0025*** | | | | ***0.0028*** | | | | |  |  |
| OXPHOS_CI+CII-linked_ | 51.5 | (45-57) | | | 36.5 | (27-41) | | 31.5 | | (29-36) | | | 0.0026 | | ***0.0043*** | | | | ***0.0006*** | | | | 0.2711 | | | | |  |  |
| ETS_CI+CII_ | 47.5 | (45-52) | | | 37.5 | (31-45) | | 32.0 | | (29-41) | | | 0.0316 | | ***0.0456*** | | | | ***0.0050*** | | | | 0.1870 | | | | |  |  |
| ETS_CII_ | 13.0 | (11-16) | | | 11.5 | (9-12) | | 12.0 | | (11-13) | | | 0.2916 | |  | | | |  | | | |  | | | | |  |  |
| CIV-linked | 94.5 | (81-101) | | | 70.5 | (64-81) | | 47.5 | | (42-54) | | | 0.0001 | | ***0.0310*** | | | | ***<.0001*** | | | | ***0.0038*** | | | | |  |  |

**Supplementary Figure 1 – Western Blot Images**

Citrate synthase antibodies with glyceraldehyde-3-phosphate dehydrogenase (GAPDH) used as a housekeeping protein demonstrating the protein signal in the three groups in snap frozen brain tissue.

**
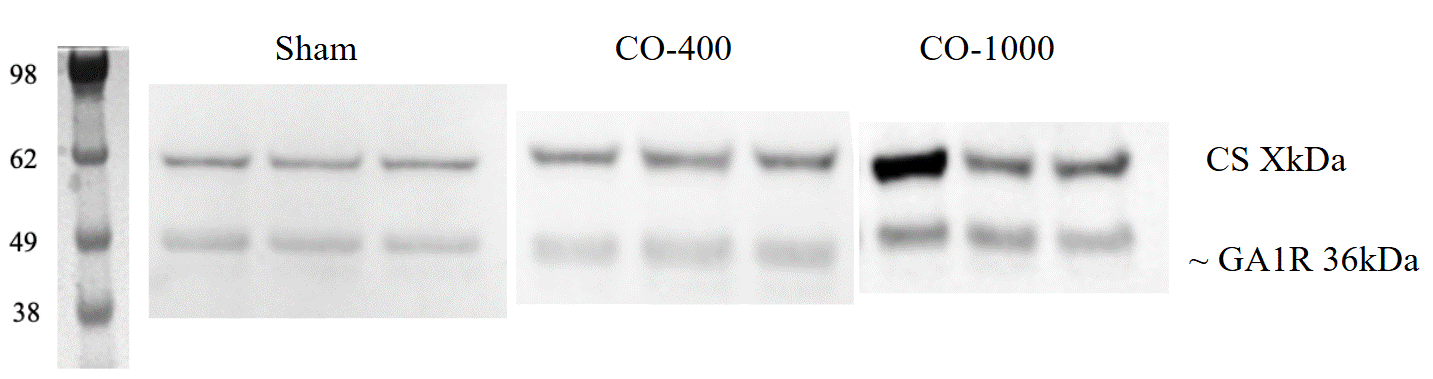
**
